# Supplementary material for: Recyclable Self-Healing Polyurethane Cross-Linked by Alkyl Diselenide with Enhanced Mechanical Properties
Source: Polymers (Basel). 2019 May 1;11(5):773. doi: 10.3390/polym11050773 (PMC6572199; doi:10.3390/polym11050773)
Supplement: Supplementary file 1 [file polymers-11-00773-s001.pdf]

Self-healing Polyurethane Cross-linked by Alkyl Diselenide with Enhanced Mechanical Properties

Yuqing Qian[1], Xiaowei An[1], Xiaofei Huang[1,2], Xiangqiang Pan[1*], Jian Zhu[1*], and Xiulin Zhu[1,3]

[1] State and Local Joint Engineering Laboratory for Novel Functional Polymeric Materials, Jiangsu Key Laboratory of Advanced Functional Polymer Design and Application, Department of Polymer Science and Engineering, College of Chemistry, Chemical Engineering and Materials Science, Soochow University, Suzhou 215123, China.

[2] Jiangsu Litian Technology Co. Ltd., Rudong County, Jiangsu 226407, China

[3] Global Institute of Software Technology, No 5. Qingshan Road, Suzhou National Hi-Tech District, Suzhou 215163, China.

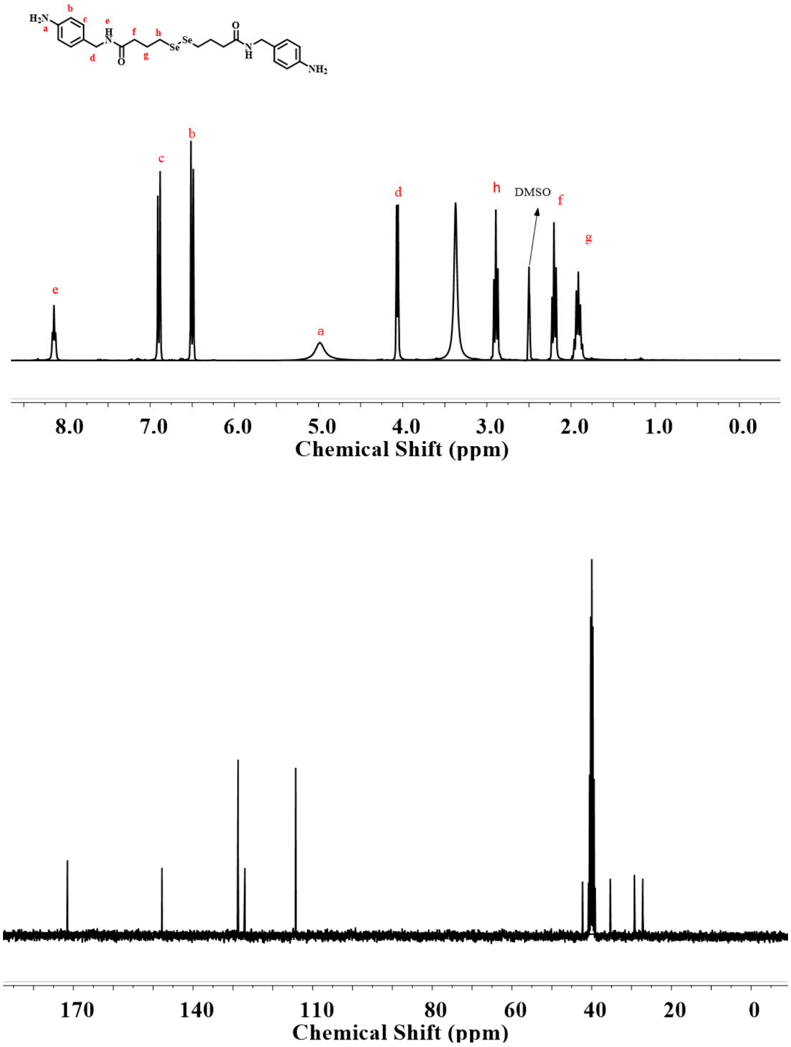

Figure S1. ^1H and ^{13}C NMR spectra recorded for diselenide functionalized diamine cross-linker in DMSO

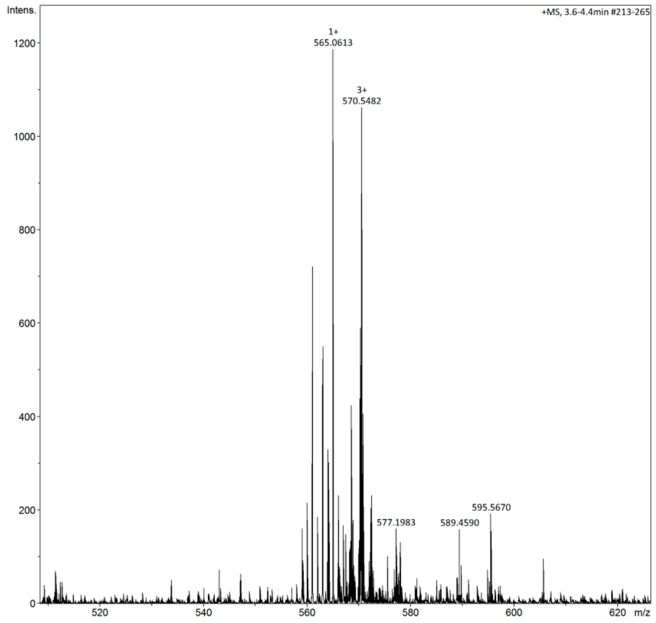

Figure S2. LC-MS spectra recorded for diselenide functionalized diamine cross-linker.

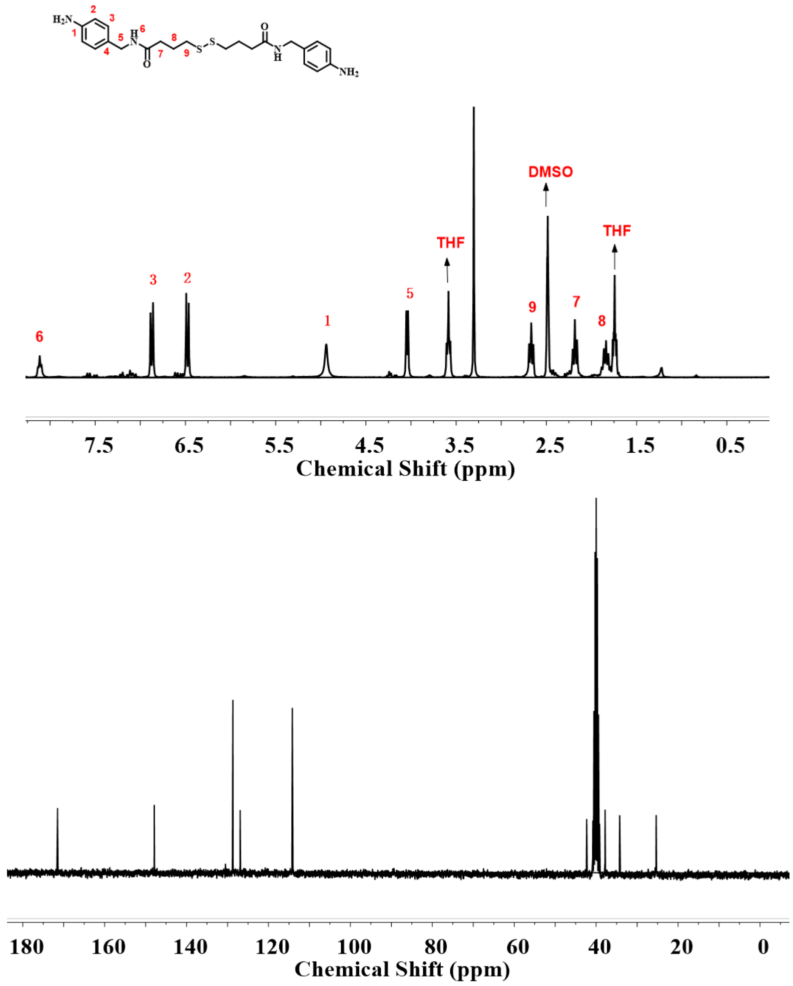

Figure S3. ^{1}H and ^{13}C NMR spectra recorded for disulfide functionalized diamine cross-linker in DMSO

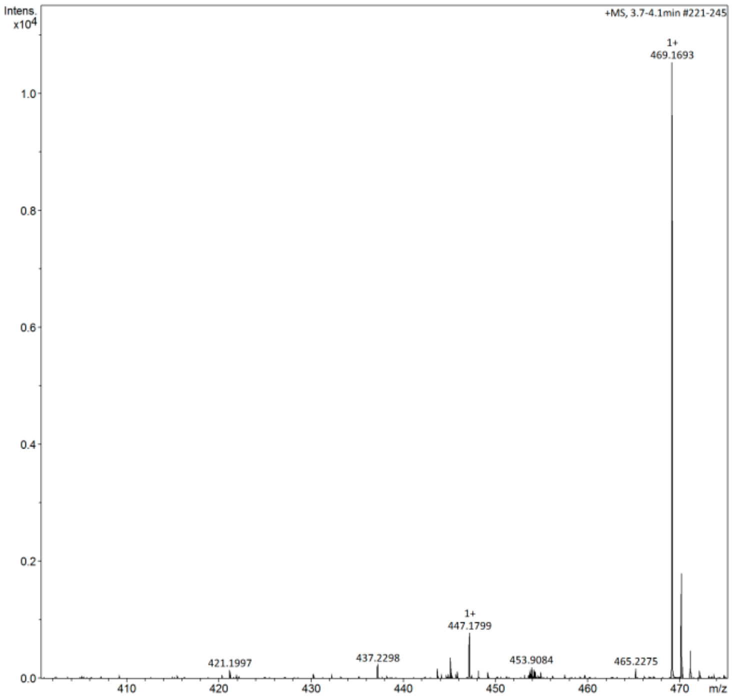

Figure S4. LC-MS spectra recorded for disulfide functionalized diamine cross-linker

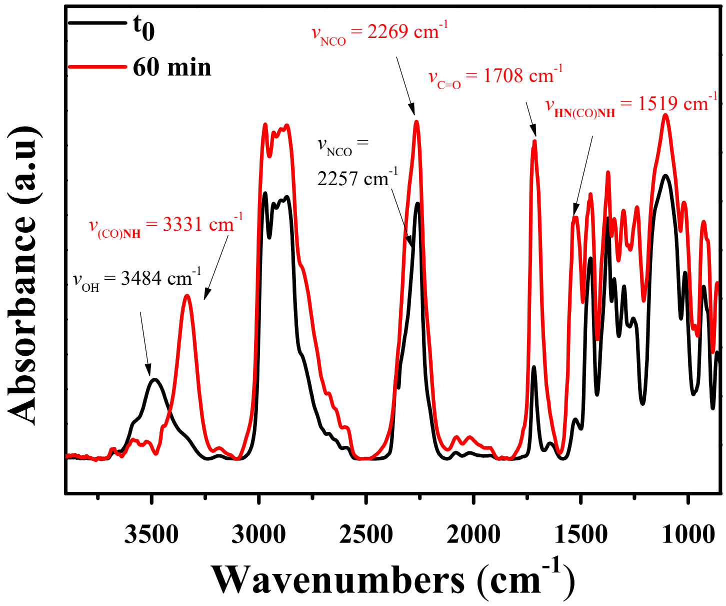

Figure S5. FTIR spectra of PPG 2 and IPDI at 70 °C at t = 0 (black trace) and t = 60 min(red trace) .

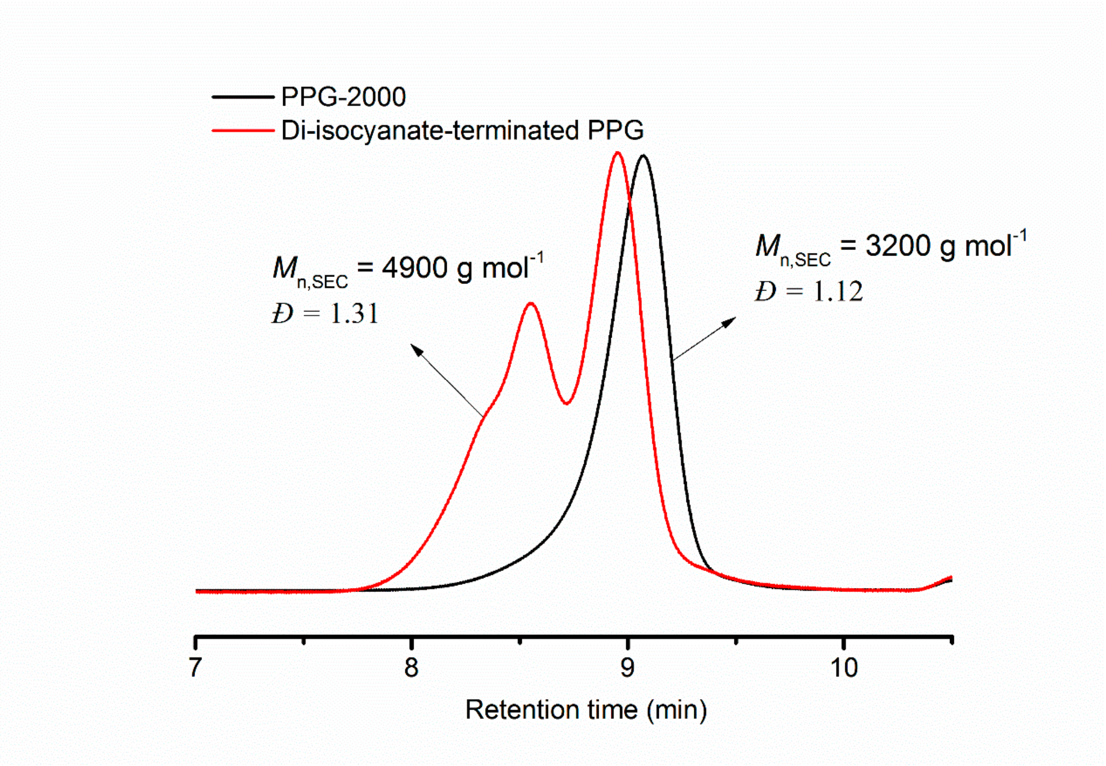

Figure S6. Curves of PPG-2000 and di-isocyanate-terminated PPG.

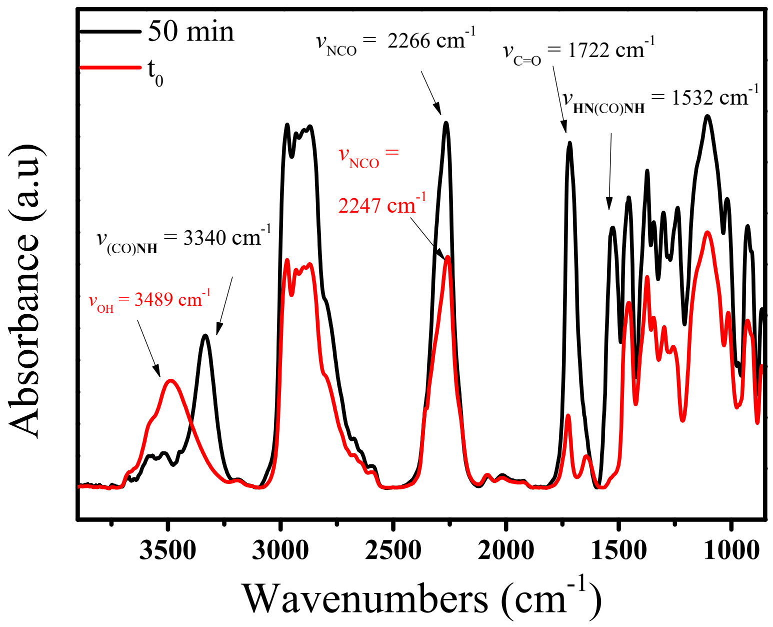

Figure S7. FTIR spectra of PPG 3 and IPDI at 70 °C at t = 0 (red trace) and t = 50 min (black trace)

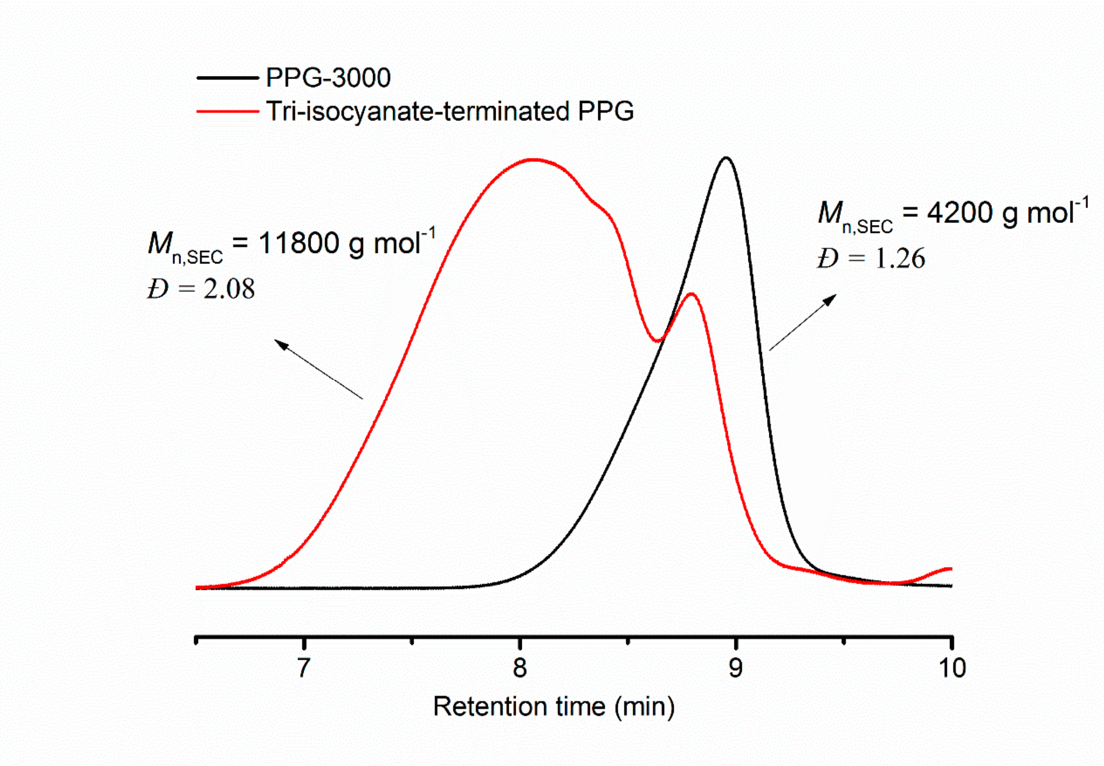

Figure S8. Curves of PPG-3000 and tri-isocyanate-terminated PPG.

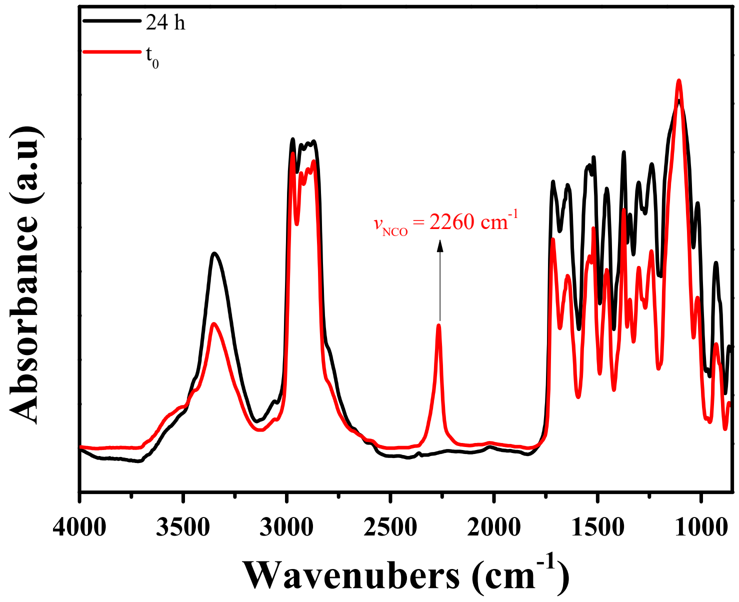

Figure S9. FTIR spectra recorded for the synthesis of alkyl diselenide based polyurethanes

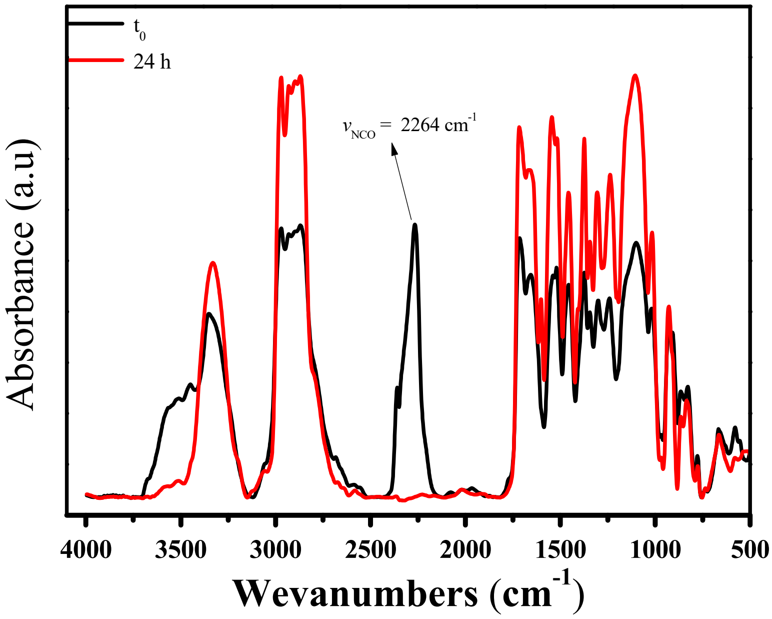

Figure S10. FTIR spectra recorded for the synthesis of alkyl disulfide based polyurethanes

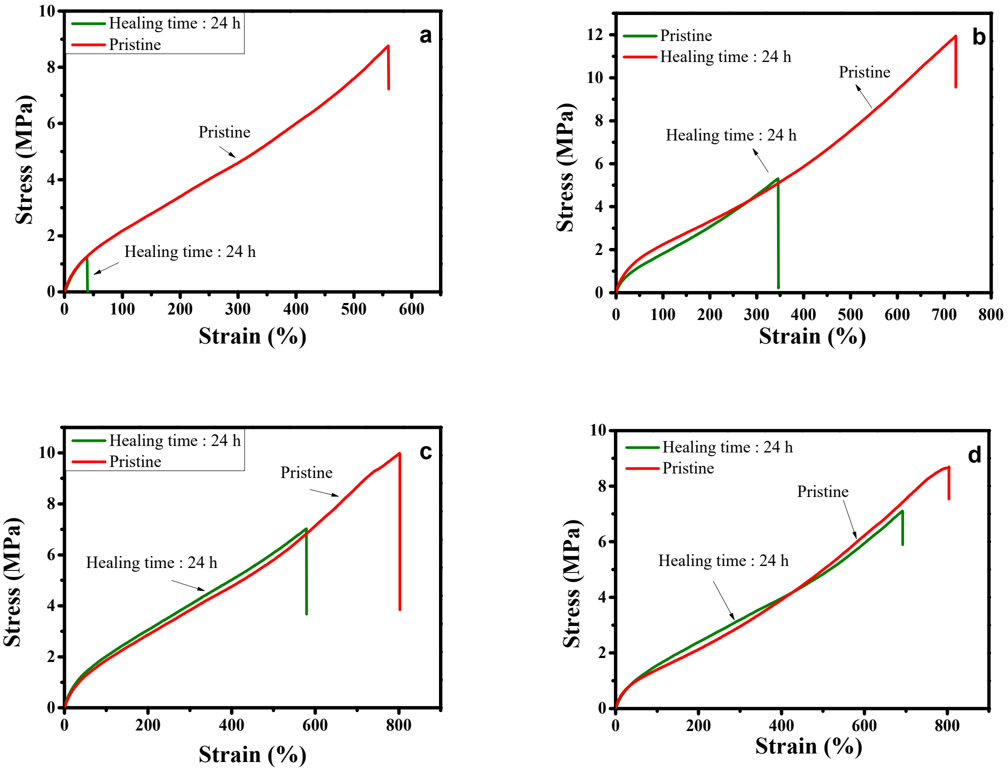

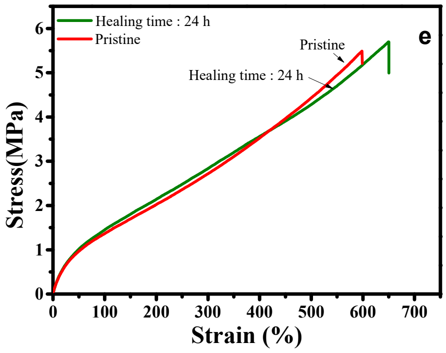

Figure S11. Representative stress-strain for five materials synthesized from different proportions of di- and tri- isocyanate-terminated PPG, a, b, c, d, e corresponds to five different materials respectively that the ratio of di-isocyanate-terminated PPG to tri-isocyanate-terminated PPG is 0: 1, 0.25: 1, 0.5: 1, 0.75: 1, and 1: 1. The healed specimens were tested after being cut in two parts, put in close contact and heal for 24 h at 25 °C.

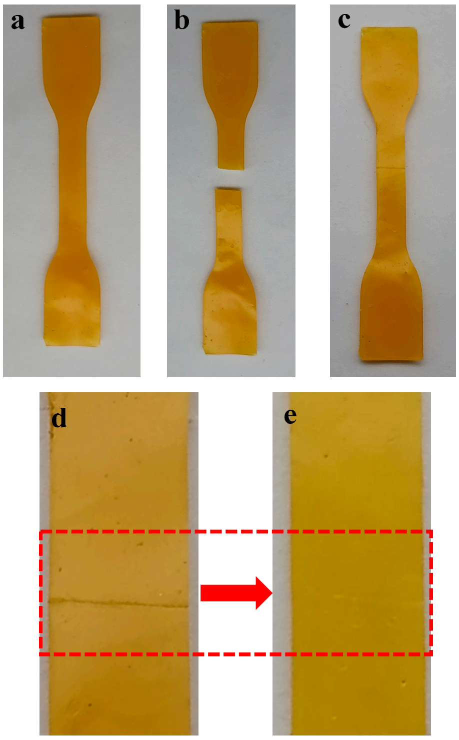

Figure S12. Self-healing process of the materials at 25 °C for 1 h. The dumbbell-shaped specimens (a) were cut in two parts (b), put in close contact (c) and heal for 1 h at 25 °C. (d) and (e) show the images of the cut-connected samples and the cut-healed samples respectively.
